# Economic evaluation of anti-CD19 CAR T-cell pathway for large B-cell lymphomas in the real-life setting: the experience of an Italian hub center in the first three years of activity

**DOI:** 10.1007/s00277-024-05766-0

**Published:** 2024-05-02

**Authors:** Rossana Di Staso, Beatrice Casadei, Marianna Gentilini, Serafina Guadagnuolo, Cinzia Pellegrini, Alessandro Broccoli, Davide Gori, Riccardo Masetti, Vittorio Stefoni, Francesca Bonifazi, Pier Luigi Zinzani, Lisa Argnani

**Affiliations:** 1 Dipartimento Di Scienze Mediche E Chirurgiche (DIMEC), Università Di Bologna, Via Massarenti, 9–40138 Bologna, Italy; 2 IRCCS Azienda Ospedaliero-Universitaria Di Bologna, Istituto Di Ematologia “Seràgnoli”, Bologna, Italy; 3 Dipartimento Di Scienze Biomediche E Neuromotorie (DIBINEM), Università Di Bologna, Bologna, Italy; 4 Pediatric Oncology and Hematology, IRCCS Azienda Ospedaliero Universitaria Di Bologna, Bologna, Italy; 5 IRCCS Azienda-ospedaliero Universitaria Di Bologna, Bologna, Italy

**Keywords:** Economic evaluation, CAR T-cell pathway, Large B-cell lymphomas, Real-life, Intention-to treat

## Abstract

Poor literature report actual and detailed costs of chimeric antigen receptor (CAR) T-cell pathway in a real-life setting. We retrospectively collect data for all patients with relapsed/refractory aggressive large B-cell lymphoma who underwent leukapheresis between August 2019 and August 2022. All costs and medical resource consumption accountability were calculated on an intention-to-treat (ITT) basis, starting from leukapheresis to the time when the patient (infused or not) exited the CAR T-cell pathway for any reason. Eighty patients were addressed to leukapheresis and 59 were finally infused. After excluding CAR-T product cost, the main driver of higher costs were hospitalizations followed by the examinations/procedures and other drugs, respectively 43.9%, 26.3% and 25.4% of the total. Regarding costs of drugs and medications other than CAR T products, the most expensive items are those referred to AEs, both infective and extra-infective within 30 days from infusion, that account for 63% of the total. Density plot of cost analyses did not show any statistically significant difference with respect to the years of leukapheresis or infusion. To achieve finally 59/80 infused patients the per capita patients without CAR-T products results 74,000 euros. This analysis covers a growing concern on health systems, the burden of expenses related to CAR T-cell therapy, which appears to provide significant clinical benefit despite its high cost, thus making economic evaluations highly relevant. The relevance of this study should be also viewed in light of continuously evolving indications for this therapy.

**Supplementary information:**

The online version contains supplementary material available at 10.1007/s00277-024-05766-0.

## Introduction

Chimeric antigen receptor (CAR) T-cells therapies represent a new class of cancer immunotherapies that genetically engineer patient T-cells to target their disease. Results from CAR T-cells clinical trials and real-life evidence have shown high rates response and durable remissions and meaningful overall survival benefits in relapsed/refractory large B-cell lymphomas (LBCL) [1]. After tisagenlecleucel (tisa-cel) and axicabtagene ciloleucel (axi-cel) were approved by FDA in 2017, these anti-CD19 CAR T products have become commercially available in Italy at the end of 2019 as novel therapeutic approaches for patients with B-cell malignant neoplasms who are refractory or relapsed after 2 or more lines of standard therapy.

Despite the reported clinical benefit, these therapies are priced amongst the most expensive cancer therapies to date [2]. In addition to direct costs of acquisition and infusion of CAR T-cells, lymphodepletion, outpatient visits and exams, there are also costs attributable to bridging therapy (BT), hospitalization, intensive care unit (ICU) admissions, laboratory activity, imaging studies, specialized and multidisciplinary teams work and management of both potentially life-threatening and mild to severe adverse events (AEs). Formal assessment of these aspects will improve knowledge about this new therapeutic approach, effective economic evaluation and understanding its actual cost.

From a scientific literature review it has been found that most (and poor) research assessing the costs of CAR T-cell therapies consider only infused patients in calculating the total expenditure of the patient path [3, 4]. This would lead to a bias in the estimates as it would exclude a key part of the CAR T-cell patient cohort. For this purpose, we believe that the intention-to-treat (ITT) population, defined as comprising all patients who underwent leukapheresis regardless of whether or not they finally received CAR T-cells infusion, has to be considered in this type of analyses since if the patients had not been candidates for the CAR T-cells therapy they would not have started the path. Other research was focused on cost effectiveness or cost utility, thus considering also in these cases only infused patients [5–8].

To date, scarce research has quantified real and ITT costs attributable to CAR T-cell therapy especially in Italy where it is absolutely absent and country-specific economic evaluations are necessary to determine whether and how to offer patients these highly personalized forms of immunotherapy [2]. In Italy, only a process mapping and activity-based costing methodologies were applied to collect the hospital costs related to CAR T-cells pathway on 47 patients (only infused patients) [9]. Here we report actual costs sustained for the first 80 LBCL patients set off on the path with the first approved indication, i.e. third line (regardless of whether they finally infused or not) in a big Italian hub center starting from tisa-cel and axi-cel commercialization in 2019 to October 2022. This present research will cover an important knowledge gap, i.e. the real economic impact of CAR T-cell pathway.

## Methods

### Patients

LBCL patients scheduled for CAR T-cell therapy (axi-cel or tisa-cel) who underwent leukapheresis between August 2019 and August 2022 referred to the hub center of Bologna (IRCCS Azienda Ospedaliero-Universitaria di Bologna, Italy) were considered for this cost analysis on ITT basis. Patients’ data were recorded from the day of leukapheresis until our data cut-off set at 31 October 2022 (considering patients who had at least one response assessment after CAR T-cell infusion, i.e. one-month follow-up). For patients who were not infused, the date on which physicians made the decision not to infuse was used as the date of exit from the CAR T-cell pathway and no other costs since that date have been charged to the patient as a CAR T-cell pathway cost. Reasons for non-infusion may comprise progression of disease (mainly in central nervous system), patient refusal, infections, complete response achieved with BT, psychiatric disorder and insufficient apheresis product; patients who relapsed after or were refractory to CAR T-cell infusion and who started another therapy were censored at the day the new therapy was scheduled.

### Study design

Overall, CAR T-cell pathway costs include all the exams, therapies, hospitalizations and personnel time from leukapheresis until to patient exited from CAR T-cell pathway due to any cause: decision to not infuse, death, progression, further anti-lymphoma therapy or last available follow up, whichever came first or as applicable.

To make the cost analysis easier to read and more informative, four stages were identified within patient’s journey:

- Stage 1: From the day of (first) leukapheresis was done to the day before admission for infusion.
- Stage 2: From the first day of admission until day + 30.
- Stage 3: From day + 31 until day + 180.
- Stage 4: From the day + 181 until 1 year following infusion.

The study was approved by our institutional board (Ethical Committee AVEC of Bologna, approval id 1043/2021/Oss/AOUBo) under a specific project of the University of Bologna (ALMA IDEA 2022 CUP:J33C22001420001). All participants gave written informed consent (when applicable) in accordance with the Declaration of Helsinki to retrospectively collect their data. As for the retrospective design of the study, we received an authorization to analyze data also of patients who were deceased or lost to follow up at the time of data capturing.

### Exams packages

Packages, check-ups, medical examinations and exams/procedures were constructed for each of the time-points of interest of the CAR T-cell pathway (Table S1-S2). To each exam/procedure constituting these latter packages was assigned the price captured from the regional tariff schedule of Emilia Romagna updated as October 2022 to have a homogeneity of costs and to be able to compare annual expenses without the potential bias of inflation [10]. For convenience, examinations/clinical procedures were divided into four categories, namely hematological exams (including the cost of blood sampling), neurological exams, imaging exams related to lymphoma assessment and “other” which comprises central venous catheter placement, electrocardiogram and echocardiogram. In addition, each time point was assigned to one of the four stages. More detailed information about the time points and their stages, examination procedures packages, their costs and codes can be found in the supplementary material (Table S1-S2).

As the cohort consists of patients that were treated before the start of pandemic and in different moment of the sanitary emergency, i.e. under different emergency protocols involving different procedures with their associated costs, COVID19-related costs were not considered for this work to avoid bias in actual CAR T-cell pathway cost estimation.

### Therapies

The following therapies were considered: BT, lymphodepletion, supportive care, transfusion bags (even if are considered as therapeutic procedures, their costs were calculated separately to have a clearer vision of how they can influence the total expenses), therapies for prophylaxis and AEs, and CAR T-cell products. To estimate therapy drug costs, the price of each medication with doses and how many milligrams of product are contained in the single packet purchased were requested from the Pharmacy Department. Subsequently, based on the days of the therapy duration or the duration of the AE for which the therapy was prescribed, and the available total doses, drug costs were estimated. When precise treatment doses were not available from the records, the average of the administrable dose range in milligrams were used, multiplied, where necessary, by the adjusted weight in kilograms of the patients or their body surface area value (Haycock’s formula) taken from patient clinical records. For BT and lymphodepletion we used the actual schedule. For drugs prescribed and taken autonomously by the patient, the calculation of packages was done by rounding up, while for drugs administered in both outpatient and inpatient settings, the cost per unit was calculated starting from price provided by the Pharmacy Department. Where necessary, the cost of the outpatient staying, hospital pharmacy staff person-cost involved for compounding, nurse for administration and hospital supplies has been added. Each therapy was subsequently assigned to the belonging stage. For prophylactic therapies it was not possible to identify the specific stage, which is why it is presented only as a separate total entry (Table 1).

**Table 1:** Costs (euros) by stages

	Stage 1		Stage 2		Stage 3	Stage 4		TOTAL
	Infused (*n* = 59)	Not infused (*n* = 21)	Infused (*n* = 59)	Not infused (*n* = 21)	Infused (*n* = 43)	Infused (*n* = 18)	Not attributable to any stage	
Time/person (total)	**36,525**	**12,182**	**9,798**	**2,252**	**1,107**	**269**	**3,373**	**65,509**
*Specialized physicians*	*22,652*	*7,786*	*1,224*	*711*	*1,107*	*269*	NA	*33,752*
*Labs staff*^*a*^	*13,872*	*4,395*	*8,574*	*1,540*	NA	NA	NA	*28,382*
*Monthly meeting*	NA	NA	NA	NA	NA	NA	*3,373*	*3,373*
Exams (total)	**236,686**	**49,668**	**627,319**	**11,391**	**187,223**	**33,256**	**NA**	**1,145,545**
*Blood exams*	*40,302,52*	*11,578,36*	*492,582,50*	*10,193*	*60,733,20*	*3,425*	NA	*618,815*
*Neurology*	*20,669*	*1,196*	*35,866*	NA	*15,963*	*6,682*	NA	*80,378,25*
*Imaging exams for disease evaluation*^*b*^	*168,567*	*35,201*	*84,263*	*258*	*108,393*	*23,148*	NA	*419,832*
*Other*^*c*^	*7,147*	*1,692*	*14,607*	*939*	*2,132*	NA	NA	*26,519*
Transfusions	**12,055**	**NA**	**62,909**	**NA**	**22,643**	**NA**	**NA**	**97,608**
Hospitalization (in- and outpatient)	**97,823**	**18,769**	**1,628,788**	**14,988**	**153,627**	**NA**	**NA**	**1,913,996**
Apheresis	**24,573**	**6,848**	**NA**	**NA**	**NA**	**NA**	**NA**	**31,422**^**d**^
Total medications	**143,203**	**64,863**	**14,117,965**	**1,410,615**	**71,018**	**NA**	**168,172**	**15,975,837**
*Prophylaxis*	NA	NA	NA	NA	NA	NA	*168,172*	*168,172*
*CAR T-cells*	NA	NA	*13,494,132*	*1,375,402*	NA	NA	NA	*14,869,534*
*Medication without CAR T products*	*143,203*	*64,863*	*623,832*	*35,212*	*71,018*	NA	*168,172*	*1,106,303*
Total	**550,867**	**152,332**	**16,446,781**	**1,439,246**	**435,619**	**33,525**	**171,545**	**19,229,919**
Total without CAR T products								**4,360,384**

^a^Comprises laboratory staff, CAR T specialist, helathcare workers and pharmacists. ^b^Include only imaging exams for disease evaluation. ^c^Comprises central venous catheter placement, electrocardiogram and echocardiogram. ^d^One patients did 3 leukapheresis in total (failing two), another one did 2 leukapheresis (failing one)

*CAR* = chimeric antigen receptor; *NA* = not applicable

Only medical direct costs from the healthcare provider perspective were analyzed meaning that discount rate and initial early access programs for CAR T-cell technology, which accommodate lower prices or even free of charge, were not considered. We used the current cost of 2023 provided by our hospital Pharmacy Department as the drugs at the time of writing are no longer considered innovative (i.e. no longer paid with the formula “Payment by result”), thus data could be more informative for further economic estimates.

### Time-person

Nurses, clinicians, healthcare worker, laboratory staff, and CAR T specialists were asked about the time taken in the procedures pertaining to the whole therapeutic pathway, to estimate the procedures ranging from the preparation of the bags prior to leukapheresis to the sending of the credit note for non-infused patients and the updating of the pharmacovigilance portal of the Italian Agency for Drugs (AIFA). Having time spent and the salaries of the specific profession the cost-per-person were estimated. For transfusion service, neurology and radiotherapy departments the prices of aphaeretic/neurological/radiotherapy visits instead of cost-per-person was used [10]. A stage was assigned to each activity performed, and total costs were calculated for each patient based on his/her journey duration. For the monthly apheresis meetings that could not be charged to the individual patient, the cost of total months comprised in the study period, i.e. 37 months, was calculated.

**Fig. 1:**
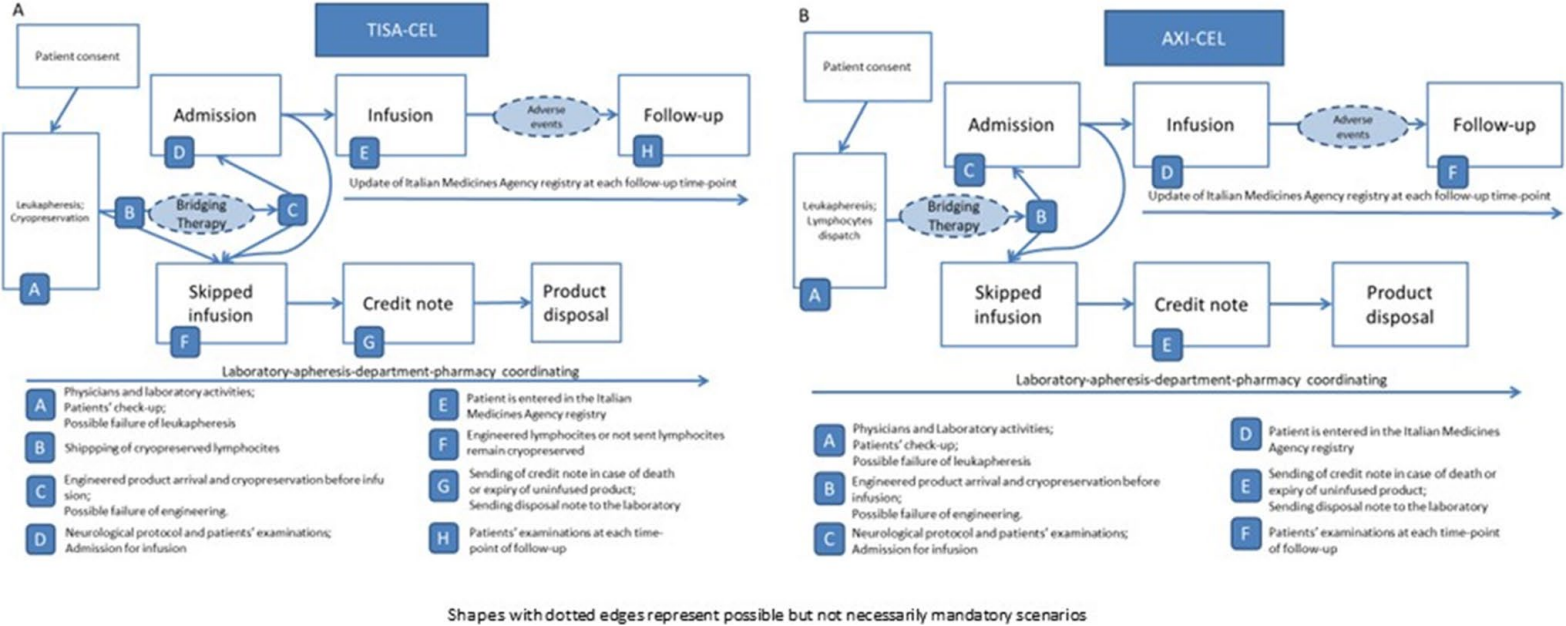
CAR T-cell pathways. (**A**) Tisagenlecleucel (tisa-cel). (**B**) Axicabtagene ciloleucel (axi-cel)

The cohort of patients considered includes patients who were infused with (or scheduled for) either tisa-cel or axi-cel. The two products have different laboratory procedures which were considered separately: a summary of the differences between the two products is provided as well as the whole pathway considered for cost estimation are provided in Fig. 1 and the descriptions of procedures, estimated time and hourly wages of professionals are available in the supplementary material (Tables S3-S4).

### Hospitalizations

For each patient a record was kept of the lengths of hospitalization in the ICU and in the other inpatient wards (hematology and transplantation departments). By comparing the start and end dates of each admission for each patient with the dates of apheresis, infusion and exit from the CAR T-cell pathway, it was possible to ascribe each hospitalization to each of the 4 stages. Once the total days of admissions for each stage had been obtained, these were multiplied by the inpatient daily costs according to the type of hospital department. In addition, when BT was administered in an outpatient setting, the sum of the costs of the individual days of access to the facility was considered (Table S4).

### Assembling data and statistical analysis

Knowing therapies, examination packages, hospitalization days, and all possible treatment-related expenses, it was possible to estimate the per-patient total costs and to construct a dataset containing all the costs listed above. As a consequence, it was possible to determine the total cost for categories, stages and time-points. Then, by dividing the total categories and stages costs by the number of patients who reached each time-point of interest, the per capita cost was obtained.

An explorative time-dependent density analysis of the costs was also performed. Kruskal–Wallis rank sum test was applied to check whether differences in costs between different years (considering both the date of leukapheresis and the date of infusion) were statistically significant. Plotted total costs includes costs for transfusion bags, hospitalizations, costs for BT, costs for prophylaxis and steroids, and costs of therapies for AEs that occurred within or after 30 days from infusion. In the density plot, costs related to patients that underwent leukapheresis in 2019 were not considered since this sub-group would be composed by only two patients. Other costs, such as costs for examinations at time-point and costs related to time-person, were not considered since they are established by hospital specific standard operation procedures (SOPs) and the aim of this additional analysis was to highlight possible difference in costs that are related to event that could change depending on physician's clinical experience, e.g. which kind of therapies should be chosen for a specific AE, the duration of both treatments and hospitalizations. Time to point events were estimated by the Kaplan–Meier method.

Data manipulation and table construction were made with v.4.2.2. of RStudio statistical software and P values for statistical significance was set at 0.05.

## Results

### Patients and outcomes

Eighty LBCL patients underwent leukapheresis during the study period and, finally, 59 ones were infused whereas 21 ones were not. Reasons for non-infusion comprised progression of disease (*n* = 12, 7 of whom in central nervous system), patient refusal (*n* = 4), insufficient apheresis product (*n* = 2), COVID19 infection (*n* = 1), complete response (*n* = 1) and psychiatric disorder (*n* = 1). The median age at leukapheresis was 57.5 years (range 20–70), and 48 patients (6003%) were males. Most were refractory to last treatment (81.3%) and all patients were heavily pre-treated, with a median number of previous lines of therapy of 3 (range 2–7). The median follow-up in the whole ITT population was 16 months (95% confidence interval 13.8–21.8). The median time from leukapheresis to infusion was 42 days (no difference between the two products occurred) with an overall response rate of 71% and an ITT median progression-free and overall survival of 7.9 months and 14.9 months, respectively.

### Stages and timepoints

Considering the 4 stages, for the ITT population the per capita cost results 240,373 euros with CAR T costs and 54,504 euros excluding CAR T products; these costs comprise prophylaxis and time-person cost for monthly meetings which cannot be ascribed to a specific stage (Table 1). Considering that finally 59/80 patients were infused, to reach this result the per capital cost (total without CAR T products/59) for successful infusions was 74,000 euros. Hospitalizations are the expense items that weigh the most on the therapeutic pathway followed by the examinations/procedures and other drugs, respectively 43.9%, 26.3% and 25.4% of the total (4,360,384 euros spent without CAR T products). For the 59 infused patients the per capita cost of transfusion bags was 1654 euros (3.8% of the total).

As the CAR T-cell pathway has specific SOPs, we were able to identify precise examinations and procedures for time point (Table S1-S2) and to calculate their costs (Table 2). Besides procedures performed during hospitalization, the higher per capita costs resulted at the day before ICU admission the follow-up time-points, namely at 3 months and 12 months after infusion, i.e. 1,961, 2,105 and 1,847 euros respectively, when both the disease assessment and the neurology package are repeated. The mean per capita costs of exams for an immune effector cell-associated neurotoxicity syndrome of grade equal or higher than 2 (immune effector cell-associated neurotoxicity syndrome [ICANS], excluding costs of hospitalization) resulted in 1,138 euros whereas for cytokine release syndrome (CRS) 271 euros. To finally achieved 59 infusions out of 80 CAR_T scheduled patients, the per capita—excluding CAR T—product was 74,000 euros.

**Table 2:** Costs (euros) of the examinations/procedures for each time-point of the CAR T-cell pathway

	Infused patients who reached the time-point, *n*	Not Infused patients who reached the time-point, *n*	Total exams	Per capita	Blood exams	Neurology	Imaging^a^	Other
Before leukapheresis	59	21	**134,349**	*1,679*	24,609	0	104,368	5,372
Before BT	45	10	**10,023**	*182*	10,023	0	0	0
Before ICU admission	59	5	**125,536**	*1,961*	1,568	21,865	98,796	3,305
Every day of hospitalization	59	5	**526,284**	*8,223*	511,341	0	9,660	5,283
Before lymphodepletion	59	5	**33,594**	*569*	22,689	0	0	10,905
1st day after infusion	59	NA	**2,433**	*41*	0	2,433	0	0
3 days after infusion	59	NA	**2,433**	*41*	0	2,433	0	0
7 days after infusion	58	NA	**2,392**	*41*	0	2,392,50	0,00	0,00
10 days after infusion	57	NA	**2,351**	*41*	0	2,351	0	0
15 days after infusion	57	NA	**2,351**	*41*	0	2,351	0	0
21 days after infusion (or at discharge)	57	NA	**10,848**	*190*	10,848	0	0	0
1 month after infusion	56	NA	**82,785**	*1,478*	10,769	0	72,016	0
3 months after infusion	43	NA	**90,526**	*2,105*	8,183	15,963	66,379	0
6 months after infusion	32	NA	**48,896**	*1,528*	6,090	0	41,152	1,653
12 months after infusion	18	NA	**33,256**	*1,847*	3,425	6,682	23,148	0
CRS^b^	50	NA	**13,578**	*271*	9,266	0	4,312	0
ICANS2^b^	21	NA	**23,903**	*1,138*	0	23,903	0	0
Total			**1,145,545**	*21,381*	618,815	80,378	419,832	26,520

^a^Include only imaging exams for disease evaluation. ^**b**^Considering the duration of all the events occurred

*BT* = bridging therapy; *CAR* = chimeric antigen receptor; *CRS* = cytokine release syndrome; *ICANS* = immune effector cell-associated neurotoxicity syndrome; *NA* = not applicable

### Medications and therapies

Regarding costs of drugs and medications other than CAR T products, the most expensive items are those referred to AEs, both infective and extra-infective within 30 days from infusion, that account for 63% of the total (Table 3); the sum of total medications cost for infective AEs both within and after 30 days from infusion, covers the 42% of the total expense. A difference between the two products can be highlight: the per capita cost for tisa-cel for infective AEs within 30 days from infusion was 16,590 euros, whereas for axi-cel was 4,383. After the first 30 days, the situation was reversed: for tisa-cel we found 2,502 euros, whereas for axi-cel 12,583 euros, respectively. Higher per capita expenses for transfusions were found for patients who received tisa-cel, i.e. 6,809 versus 3,737 for patients who underwent axi-cel with a slightly significant correlation with cytopenia (*p* = 0.041). Nevertheless, the mean per capita cost sustained for drugs and medications for infused patients was almost the same for both CAR T products, i.e. an average of about 54,000 euros. On the other hand, for not infused patients the final per capita cost was 13,000 euros for BT.

**Table 3:** Costs (euros) of therapies and medications divided as infused and not infused patients

	Infused patients						Not infused patients		
	Tisa-cel			Axi-cel					
	Total	*n*	Per capita	Total	*n*	Per capita	Total	*n*	Per capita
BT	76,297	20	*3,814*	66,905	23	*2,908*	64,863	5	*12,972*
CAR T product	6,814,236	27	*252,379*	6,679,896	30	*222,663*	1,375,401^a^	6	*229,233*
Lymphodepletion	1,192	27	*44*	1,818	32	*56*	NA	NA	NA
Levetiracetam	3,020	27	*111*	3,948	32	*123*	NA	NA	NA
Tocilizumab	86,301	27	*3,196*	107,673	32	*3,364*	35,212^b^	8	*4,401*
Drugs for infective AEs within 30 days from infusion	82,953	5	*16,590*	65,755	15	*4,383*	NA	NA	NA
Drugs for extra infective AEs within 30 days from infusion	93,424	8	*11,678*	85,618	6	*14,269*	NA	NA	NA
Steroids	216	10	*21*	554	18	*30*	NA	NA	NA
Siltuximab	3,509	2	*1,754*	19,552	9	*2,172*	NA	NA	NA
Anakinra	3,157	1	*3,157*	27,624	6	*4,604*	NA	NA	NA
Drugs for infective AEs after 30 days from infusion	7,506	3	*2,502*	62,915	5	*12,583*	NA	NA	NA
Drugs for extra-infective AEs after 30 days from infusion	0	0	*0*	595	3	*198*	NA	NA	NA
G-CSF	53	3	*17*	143	8	*17*	NA	NA	NA
Intravenous immunoglobulins	16,511	10	*1,651*	20,803	13	*1,600*	NA	NA	NA
Platelet transfusion bags	26,160	5	*5,232*	15,742	7	*2,248*	NA	NA	NA
Packed red blood cells transfusion	28,892	17	*1,577*	26,815	18	*1,489*	NA	NA	NA
Prophylaxis, antifungal	73,781	18	*4,098*	56,698	20	*2,834*	NA	NA	NA
Prophylaxis, antiviral	16,643	26	*640*	16,384	31	*528*	NA	NA	NA
Prophylaxis, antibacterial	242	21	*11*	156	21	*7*	NA	NA	NA
Prophylaxis, PJP	325	27	*12,06*	996,72	32	*31*	NA	NA	NA
Other Prophylaxis	209	5	*41*	2,735	6	*455*	NA	NA	NA
Total (average)	**7,334,634**		***308,533***	**7,263,334**		***276,574***	**1,475,478**		***246,607***
Total without CAR T (average)	**520,398**		**56,154**	**583,438**		**53,911**	**100,076**		**17,374**

^a^Includes cost of non-infused products for which the credit note has not yet been sent and are still cryopreserved in the laboratory at the cut-off date. ^b^These costs are calculated on the basis of orders for tocilizumab packages, therefore also includes charges for unused medications as tocilizumab that is ordered about one month before CAR T infusion with a nominal request

*AE* = adverse event; *Axi-cel* = axicabtagene ciloleucel; *BT* = bridging therapy; *CAR* = chimeric antigen receptor; *G-CSF* = granulocyte colony-stimulating factor; *NA* = not applicable; *PJP* = Pneumocystis jirovecii pneumonia; *Tisa-cel* = tisagenlecleucel

### Time-person

There were personnel costs attributable directly to each stage and timepoint, depending also on the different procedures belonging to the two CAR T products we considered (Fig. 1 and Table 4). The time spent for the (bi)monthly (or as needed) apheresis meeting cannot be ascribed to a single timepoint nor estimated as a mean for patient, since it is a planning meeting independent of the number of patients who are candidates for CAR T-cell therapy; in one year, it is estimated about 1,100 euros.

**Table 4:** Costs (euros) of personnel for the CAR T-cell pathway

	Infused		Not infused				
	Tisa-cel *n* = 27	Axi-cel *n* = 32	Tisa-cel *n* = 5	Axi-cel *n* = 11	Lymphocytes never sent *n* = 4 (tisa-cel)	Failed leukapheresis *n* = 1	Total
Lab staff	13,520	8,887	2,261	2,473	1,304	88	28,535
Healthcare worker	348	640	72	220	48	12	1,341
Pharmacist	2,419	2,867	550	1,139	204	51	7,233
Physician	7,555	8,899	1,248	2,599	704	176	21,182
CAR T specialist	1,406	1,666	277	578	185	46	4,161
TOTAL	25,249	22,961	4,409	7,011	2,447	373	62,453
Per capita (patient)	935	717	881	637	611	373	1058^a^

^a^Considering 59 infused patients

*Axi-cel* = axicabtagene ciloleucel; *CAR* = chimeric antigen receptor; *Tisa-cel* = tisagenlecleucel

The total personnel costs for both products did not differ between infused (1,652 euros for each patient) and not infused patients (1,518 euros for each patient). On the other hand, when considering also time-person costs for not infused patients with lymphocytes cryopreserved and never sent (tisa-cel, as for its different pathway procedures) or time-person cost for patients who failed leukapheresis, the per capita (patient) cost is higher for not infused patients. In detail, for infused patients the personnel expense cost results 1,652 euros/patient while for not infused ones it was 2,502/patient.

### Hospitalizations

Hospitalization costs for patients who started the CAR T-cell pathway are shown in Table 5 divided as outpatient and transplantation ward, ICU and hematology ward. Outpatient ward referred to first visit, BT and follow-up after infusion. In hematology ward were counted days of hospitalization due to BT or AEs after CAR T infusion, and leukapheresis for only two patients. About 76% of not infused patients had hospitalization also in transplantation unit as the event that cause the exit from CAR T pathway occurred when the patient was already admitted in the transplantation ward for disease restaging and neurological examinations. AEs were managed in all the four settings depending on their severity and compatibly with the availability of the hospital.

**Table 5:** Hospitalization costs (euros)

	Infused				Not infused			
	Tisa-cel (*n* = 27)		Axi-cel (*n* = 32)		Correctly engineered product (*n* = 16)		Lymphocytes never sent (*n* = 4, tisa-cel)	Failed leukapheresis (*n* = 1)
	Total cost (euros)	Days of hospitalization, n	Total cost (euros)	Days of hospitalization, n	Total cost (euros)	Days of hospitalization, n	Total cost (euros)	Total cost (euros)
Outpatient ward	11,615^a^	29	27,273	41	10,776	27	0	0
Transplantation ward	664,481	665	794,379	795	22,982	23	0	0
ICU	61,184	33	157,597	85	0	NA	0	0
Hematology ward	91,675	126	72,030	99	0	NA	0	0

^a^Includes cost of daily staying in outpatient ward, cost of blood sampling and cost of nurses’ time, as applicable

*Axi-cel* = axicabtagene ciloleucel; *ICU* = intensive care unit; *NA* = not applicable; *Tisa-cel* = tisagenlecleucel

The mean length of hospitalization in transplantation ward for infused patients was 25 days (for both products). ICU hospitalization was required for 15 (25.4%) of infused patients (4 received tisa-cel and 11 received axi-cel) with a mean of 8 days, with no difference between the two products in the length of hospitalization.

### Time-dependent density analysis

**Fig. 2:**
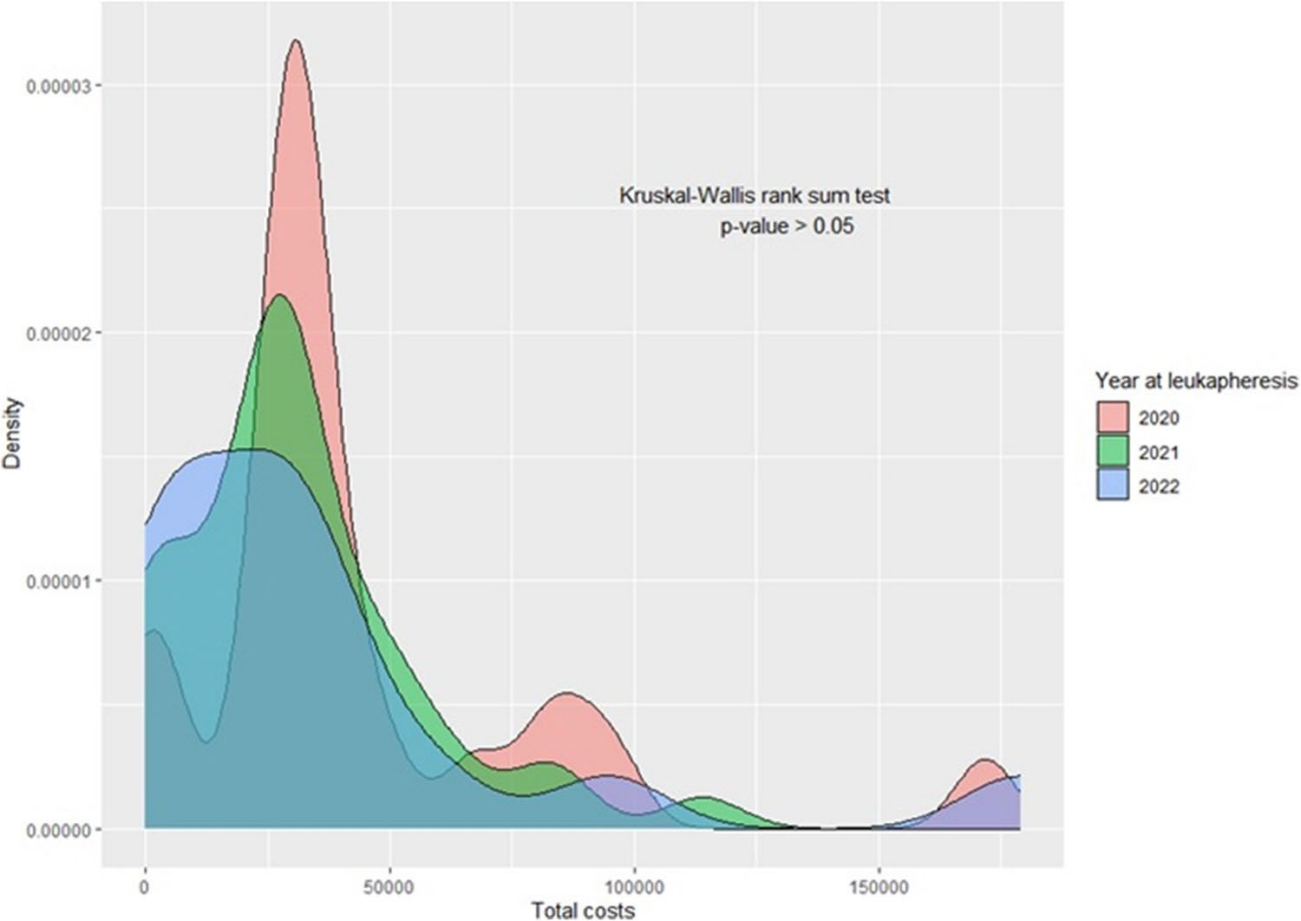
Density plot of costs per year of leukapheresis in the intention-to-treat population

Regarding the costs incurred per year, considering date of leukapheresis in 2019 we had 2 patients, in 2020 23 patients, in 2021 39 patients and in 2022 16 patients. The density of costs was explored in the ITT population (Fig. 2). No statistically significant difference in costs with respect to the years of leukapheresis occurred (detailed results are collected in Table S5). Considering only infused population, in 2019 we had 2 patients who received CAR T-cells, in 2020 20 patients, in 2021 28 ones and in 2022 9 patients, respectively: considering the year of infusion with expenditure from both leukapheresis and infusion again no significant statistical difference occurred.

## Discussion

CAR T-cell therapies are expected to bring substantial health benefits but also exposes national healthcare systems to very large expenses [3]. At the same time, an impressive increase in trial activity heralds an expansion of CAR T-cell therapies to many more indications in the near future, of which hematological cancers currently play the most significant role even if the research on CAR T-cells therapy is a rapidly developing field also in solid tumors [11]. Therefore, these therapies may have a considerable incremental budget impact on healthcare expenditures, especially in the field of hematology-oncology. Moreover, the costs associated with these therapies are not limited to acquisition costs alone. Other costs that will have a substantial impact are hospitalizations as well as other costs related to the treatment of AEs and to the multidisciplinary work that turns on this therapy.

To date, scarce literature is available about the actual costs that a public hospital has to face to when a patient is scheduled for CAR T-cells therapy. Here we presented the actual costs sustained by a big hub center in Italy throughout the first three years of experience with two CAR T-cell products for LBCL patients in the everyday clinical practice. This is not a cost effectiveness analysis, the primary goal of this research was merely descriptive to return a precise picture at our best of the effective cost of the entire therapeutic process with the limits of a retrospective data collection. To this aim, we considered all the patients on an ITT basis, i.e. we calculated all the costs from leukapheresis for each subject who started the pathway regardless of whether or not he/she finally received CAR T-cells infusion. To our knowledge this is the largest study on ITT analysis performed, and only other similar one was published even if personnel cost and all medications expenses were not considered in that report [2].

Leukapheresis represents one of the first steps in CAR T-cells therapy and was chosen as the starting point for computing the outcomes of interest because it is common to all patients. We decided to not choose the referral date or, unlike Chacim et al., the date of patient consent as, due to the intrinsic nature of a hub center, these starting points might have added bias in the calculation of costs (too variable among patients the time length from date of referral/consent and the leukapheresis) [2]. In addition, from leukapheresis to the first year of follow up, all patients go through the same pathway as we have specific SOPs which allowed us to calculate stages, time points and exams packages.

The detailed photograph that we have reported could be a starting point for improvement and to understand where the highest costs are. Our ITT analysis revealed an actual per capita cost to finally achieve 59/80 infusions of about 74,000 euros excluding CAR T products. The principal aim of this economic evaluation is to provide recommendations or suggest modifications in practice healthcare institutions. In fact, our research provides indications of where improvements can be made at both clinical and organizational level, e.g. reducing the time between leukapheresis and infusion or the prevention or a better management of AEs. For example, if cytokine release syndrome or immune effector cell–associated neurological syndrome could be prevented, this may reduce the need for tocilizumab and/or ICU admission for such severe AEs [12]. Another example is the cost-intensive need for prolonged supportive transfusions and growth factor support for patients with post- CAR-T cytopenia or for infective AEs that account for the 42% of the total medications/drugs expenses (our study cover the pathway period until 1 year after infusion) [13, 14]. In addition, identify early patients not eligible for this therapeutic pathway will lead to costs decrease, especially in medications, drugs and hospitalizations.

We also carried out an analysis by year because we had hypothesized that at the beginning of commercialization the poor confidence with this new therapy could have affected the management of patients. The results disprove this hypothesis with respect to the years of both leukapheresis and infusion, probably because our Center had previous experience with several clinical trials on CAR T-cells.

Due to the high price of CAR T-cell therapy, cost-effectiveness analysis plays a significant role in evaluating the value of the drug and providing treatment options. We make several suggestions to address the uncertainty raised earlier in the cost-effectiveness analysis of the therapy [15]. Thus, next steps will be a cost-effectiveness analysis in our real-life experience, the comparisons with other agents which share the same indication (e.g. bispecific agents) and a complete prospective collection of also the costs incurred by patients to understand the actual accessibility of this therapy and/or to provide data to other accredited structures [16, 17].

## Limitations

The study is not without limitations. Its retrospective design may have led to an underestimation of concomitant medications, and we were unable to estimate costs sustained by patients although they would have been interesting as we are a hub center.

In fact, distance and time to the nearest administering facility as well as staying near the hospital are key drivers of cost. In addition, we don't have quality of life indices [18]. Another cost lacking is the one related to the tank and its maintenance: we cannot estimate them as they are shared with other cell products.

## Conclusion

Our analysis covers a growing concern on health systems, the burden of expenses related to CAR T-cell therapy, which appears to provide significant clinical benefit despite its high cost, thus making economic evaluations highly relevant especially on ITT basis. Implementing a CAR-T program requires a huge investment, and the launch of new CAR T and/or the extension of their indications, may require additional investments. As a consequence, the relevance of this study should be viewed in light of continuously evolving indications for this therapy, but also in providing hospitals that are in the process of being accredited for these therapies the details on the costs they will incur.

### Supplementary information

Below is the link to the electronic supplementary material.

Supplementary file1 (DOCX 76 KB)

## Data Availability

The data underlying this article are available from the corresponding author upon reasonable request.

## Abbreviations

*AE*: Adverse event
*AIFA*: Italian Agency for Drugs
*axi-cel*: Axicabtagene ciloleucel
*BT*: Bridging therapy
*CAR*: Chimeric antigen receptor
*CRS*: Cytokine release syndrome
*ICANS*: Immune effector cell-associated neurotoxicity syndrome
*ICU*: Intensive care unit
*ITT*: Intention to treat
*LBCL*: Large B-cell lymphoma
*SOP*: Standard operation procedures; tisa-cel: tisagenlecleucel
