# Supplementary material for: Economic evaluation of anti-CD19 CAR T-cell pathway for large B-cell lymphomas in the real-life setting: the experience of an Italian hub center in the first three years of activity
Source: Ann Hematol. 2024 May 2;103(7):2499–509. doi: 10.1007/s00277-024-05766-0 (PMC11224113; doi:10.1007/s00277-024-05766-0)
Supplement: Supplementary file 1 — Supplementary file1 (DOCX 76 KB) [file 277_2024_5766_MOESM1_ESM.docx]

**Supplementary Methods (Tables)**

**Table S1. Exams for each time point with membership package, prices and regional fee schedule.**

| **Exams/Procedures** | **Membership package** | **Cost (euros)** | **regional tariff ID** |  | **Time points (link in S2)** | | | | | | | | | | | | | | | |
| --- | --- | --- | --- | --- | --- | --- | --- | --- | --- | --- | --- | --- | --- | --- | --- | --- | --- | --- | --- | --- |
|  |  |  |  | **1** | **2** | **3** | **4** | **5** | **6** | **7** | **8** | **9** | **10** | **11** | **12** | **13** | **14** | **15** | **16** | **17** |
| central venous catheter placement | OTHER EXAMS | 154.90 € | IH38.94.2 | 0 | 0 | 0 | 1 | 0 | 0 | 0 | 0 | 0 | 0 | 0 | 0 | 0 | 0 | 0 | 0 | 0 |
| electrocardiogram | OTHER EXAMS | 12.50 € | m189.52 | 1 | 0 | 0 | 1 | 0 | 0 | 0 | 0 | 0 | 0 | 0 | 0 | 0 | 0 | 0 | 0 | 0 |
| echocardiogram | OTHER EXAMS | 51.65 € | m188.72.1 | 1 | 0 | 0 | 1 | 1 | 0 | 0 | 0 | 0 | 0 | 0 | 0 | 0 | 1 | 0 | 0 | 0 |
| PET total body | IMAGING EXAMS | 1,286.00 € | m192.18.6 | 1 | 0 | 0 | 1 | 1 | 0 | 0 | 0 | 0 | 0 | 0 | 1 | 1 | 1 | 1 | 0 | 0 |
| Chest x-ray | IMAGING EXAMS | 18.60 € | m187.44.1 | 1 | 0 | 0 | 0 | 0 | 0 | 0 | 0 | 0 | 0 | 0 | 0 | 0 | 0 | 0 | 0 | 0 |
| CT scan (abdomen, with contrast) | IMAGING EXAMS | 88.30 € | I88.01.1 | 0 | 0 | 0 | 1 | 1 | 0 | 0 | 0 | 0 | 0 | 0 | 0 | 1 | 0 | 0 | 0 | 0 |
| CT scan (neck, with contrast) | IMAGING EXAMS | 83.15 € | m187.03.7 | 0 | 0 | 0 | 1 | 1 | 0 | 0 | 0 | 0 | 0 | 0 | 0 | 1 | 0 | 0 | 0 | 0 |
| CT scan (chest, with contrast, HR) | IMAGING EXAMS | 86.25 € | m187.81 | 0 | 0 | 0 | 1 | 1 | 0 | 0 | 0 | 0 | 0 | 0 | 0 | 1 | 0 | 0 | 0 | 0 |
| CT scan (chest, without contrast, HR) | IMAGING EXAMS | 86.25 € | m187.81 | 0 | 0 | 0 | 0 | 0 | 0 | 0 | 0 | 0 | 0 | 0 | 0 | 0 | 0 | 0 | 1 | 0 |
| CT scan (brain, with contrast, HR) | NEUROLOGICAL EXAMS | 83.15 € | as87.03 | 0 | 0 | 0 | 0 | 0 | 0 | 0 | 0 | 0 | 0 | 0 | 0 | 0 | 0 | 0 | 0 | 0 |
| Brain PET | NEUROLOGICAL EXAMS | 1,286.00 € | m192.11.6 | 0 | 0 | 0 | 0 | 0 | 0 | 0 | 0 | 0 | 0 | 0 | 0 | 0 | 0 | 0 | 0 | 0 |
| Brain magnetic resonance | NEUROLOGICAL EXAMS | 330.00 € | m188.91.2 | 0 | 0 | 0 | 1 | 0 | 0 | 0 | 0 | 0 | 0 | 0 | 0 | 1 | 0 | 1 | 0 | 1^a^ |
| Electroencephalogram | NEUROLOGICAL EXAMS | 23.25 € | m89.14 | 0 | 0 | 0 | 1 | 1 | 1 | 1 | 1 | 1 | 1 | 0 | 0 | 1 | 0 | 1 | 0 | 1 |
| Neurological examination (with neuropsychological testing) | NEUROLOGICAL EXAMS | 18.00 € | I89.01.c | 0 | 0 | 0 | 1 | 1 | 1 | 1 | 1 | 1 | 1 | 0 | 0 | 1 | 0 | 1 | 0 | 1 |
| Anti-Hepatitis B core antibodies | HAEMATOLOGICAL EXAMS | 9.70 € | m191.17.5 | 1 | 0 | 0 | 1 | 0 | 0 | 0 | 0 | 0 | 0 | 0 | 0 | 0 | 0 | 0 | 0 | 0 |
| IgM anti-HBc antibodies | HAEMATOLOGICAL EXAMS | 10.35 € | m191.18.1 | 1 | 0 | 0 | 0 | 0 | 0 | 0 | 0 | 0 | 0 | 0 | 0 | 0 | 0 | 0 | 0 | 0 |
| IgG anti HBe antibodies | HAEMATOLOGICAL EXAMS | 9.70 € | m191.17.5 | 1 | 0 | 0 | 0 | 0 | 0 | 0 | 0 | 0 | 0 | 0 | 0 | 0 | 0 | 0 | 0 | 0 |
| anti HBs antibodies | HAEMATOLOGICAL EXAMS | 9.90 € | m191.18.3 | 1 | 0 | 0 | 1 | 0 | 0 | 0 | 0 | 0 | 0 | 0 | 0 | 0 | 0 | 0 | 0 | 0 |
| IgG anti HCV antibodies | HAEMATOLOGICAL EXAMS | 9.70 € | m191.17.5 | 1 | 0 | 0 | 1 | 0 | 0 | 0 | 0 | 0 | 0 | 0 | 0 | 0 | 0 | 0 | 0 | 0 |
| Treponema Pallidum antibodies | HAEMATOLOGICAL EXAMS | 7.90 € | m191.10.2 | 1 | 0 | 0 | 1 | 0 | 0 | 0 | 0 | 0 | 0 | 0 | 0 | 0 | 0 | 0 | 0 | 0 |
| Serum electrophoresis | HAEMATOLOGICAL EXAMS | 5.00 € | m190.38.4 | 1 | 0 | 0 | 1 | 0 | 0 | 0 | 0 | 0 | 0 | 0 | 0 | 0 | 0 | 0 | 0 | 0 |
| Galactomannan | HAEMATOLOGICAL EXAMS | 3.00 € | m190.25.4 | 1 | 0 | 0 | 0 | 0 | 0 | 0 | 0 | 0 | 0 | 0 | 0 | 0 | 0 | 0 | 1 | 0 |
| Respiratory function tests | HAEMATOLOGICAL EXAMS | 24.50 € | m189.38.5 | 0 | 0 | 0 | 1 | 1 | 0 | 0 | 0 | 0 | 0 | 0 | 0 | 0 | 0 | 0 | 0 | 0 |
| CMV antibodies | HAEMATOLOGICAL EXAMS | 8.50 € | m191.14.1 | 1 | 0 | 0 | 1 | 0 | 0 | 0 | 0 | 0 | 0 | 0 | 0 | 0 | 0 | 0 | 0 | 0 |
| HIV antibodies | HAEMATOLOGICAL EXAMS | 10.55 € | m191.22.4 | 1 | 0 | 0 | 1 | 0 | 0 | 0 | 0 | 0 | 0 | 0 | 0 | 0 | 0 | 0 | 0 | 0 |
| Hematological examination | HAEMATOLOGICAL EXAMS | 23.00 € | m89.7 | 1 | 1 | 0 | 0 | 0 | 0 | 0 | 0 | 0 | 0 | 1 | 1 | 1 | 1 | 1 | 0 | 0 |
| Complete urine test | HAEMATOLOGICAL EXAMS | 3.00 € | m190.44.3 | 1 | 0 | 1 | 1 | 0 | 0 | 0 | 0 | 0 | 0 | 0 | 0 | 0 | 0 | 0 | 0 | 0 |
| Uric acid | HAEMATOLOGICAL EXAMS | 2.00 € | m190.44.1 | 1 | 1 | 1 | 1 | 0 | 0 | 0 | 0 | 0 | 0 | 1 | 1 | 1 | 1 | 1 | 1 | 0 |
| Albumin | HAEMATOLOGICAL EXAMS | 3.00 € | m190.05.1 | 1 | 1 | 1 | 1 | 0 | 0 | 0 | 0 | 0 | 0 | 1 | 1 | 1 | 1 | 1 | 1 | 0 |
| Alanine aminotransferase | HAEMATOLOGICAL EXAMS | 2.00 € | m190.04.5 | 1 | 1 | 1 | 1 | 0 | 0 | 0 | 0 | 0 | 0 | 1 | 1 | 1 | 1 | 1 | 1 | 0 |
| Pancreatic amylase | HAEMATOLOGICAL EXAMS | 6.00 € | m190.05.5 | 1 | 1 | 1 | 1 | 0 | 0 | 0 | 0 | 0 | 0 | 0 | 0 | 0 | 0 | 0 | 1 | 0 |
| Antithrombin III | HAEMATOLOGICAL EXAMS | 3.00 € | Rm190.82.1 | 1 | 0 | 1 | 1 | 0 | 0 | 0 | 0 | 0 | 0 | 0 | 0 | 0 | 0 | 0 | 0 | 0 |
| Time of activated partial thromboplastin | HAEMATOLOGICAL EXAMS | 3.00 € | m190.76.1 | 1 | 0 | 1 | 1 | 0 | 0 | 0 | 0 | 0 | 0 | 1 | 1 | 1 | 1 | 1 | 1 | 0 |
| Aspartate aminotransferase | HAEMATOLOGICAL EXAMS | 2.00 € | m190.09.2 | 1 | 1 | 1 | 1 | 0 | 0 | 0 | 0 | 0 | 0 | 1 | 1 | 1 | 1 | 1 | 1 | 0 |
| Prothrombin activity | HAEMATOLOGICAL EXAMS | 3.00 € | Rm190.82.1 | 1 | 0 | 1 | 1 | 0 | 0 | 0 | 0 | 0 | 0 | 1 | 1 | 1 | 1 | 1 | 1 | 0 |
| Direct and indirect bilirubin | HAEMATOLOGICAL EXAMS | 2.00 € | m190.10.5 | 1 | 1 | 1 | 1 | 0 | 0 | 0 | 0 | 0 | 0 | 1 | 1 | 1 | 1 | 1 | 1 | 0 |
| Total bilirubin | HAEMATOLOGICAL EXAMS | 2.00 € | m190.10.4 | 1 | 1 | 1 | 1 | 0 | 0 | 0 | 0 | 0 | 0 | 1 | 1 | 1 | 1 | 1 | 1 | 0 |
| Calcium | HAEMATOLOGICAL EXAMS | 2.00 € | m190.11.4 | 1 | 1 | 1 | 1 | 0 | 0 | 0 | 0 | 0 | 0 | 1 | 1 | 1 | 1 | 1 | 1 | 0 |
| Chlorine | HAEMATOLOGICAL EXAMS | 2.00 € | m190.13.3 | 0 | 1 | 1 | 1 | 0 | 0 | 0 | 0 | 0 | 0 | 0 | 0 | 0 | 0 | 0 | 1 | 0 |
| CMV DNA | HAEMATOLOGICAL EXAMS | 36.15 € | R91.15.2 | 1 | 0 | 1 | 1 | 0 | 0 | 0 | 0 | 0 | 0 | 1 | 1 | 1 | 1 | 1 | 1 | 0 |
| Total CO2 + excess of bases + pCO2 + pO2 (hemogas-analysis) | HAEMATOLOGICAL EXAMS | 20.45 € | m189.66 | 0 | 0 | 1 | 1 | 0 | 0 | 0 | 0 | 0 | 0 | 0 | 0 | 0 | 0 | 0 | 0 | 0 |
| Platelet count | HAEMATOLOGICAL EXAMS | 2.00 € | m190.71.3 | 1 | 1 | 1 | 1 | 0 | 0 | 0 | 0 | 0 | 0 | 1 | 1 | 1 | 1 | 1 | 1 | 0 |
| Creatine kinase | HAEMATOLOGICAL EXAMS | 2.00 € | m190.15.4 | 0 | 1 | 1 | 1 | 0 | 0 | 0 | 0 | 0 | 0 | 0 | 0 | 0 | 0 | 0 | 0 | 0 |
| Creatinine | HAEMATOLOGICAL EXAMS | 2.00 € | m190.16.3 | 1 | 1 | 1 | 1 | 0 | 0 | 0 | 0 | 0 | 0 | 1 | 1 | 1 | 1 | 1 | 1 | 0 |
| D-Dimer | HAEMATOLOGICAL EXAMS | 9.00 € | m190.61.4 | 1 | 0 | 1 | 1 | 0 | 0 | 0 | 0 | 0 | 0 | 0 | 0 | 0 | 0 | 0 | 0 | 0 |
| Haemochromocytmetric examination | HAEMATOLOGICAL EXAMS | 4.00 € | m190.62.2 | 1 | 1 | 1 | 1 | 0 | 0 | 0 | 0 | 0 | 0 | 1 | 1 | 1 | 1 | 1 | 1 | 0 |
| Ferritin | HAEMATOLOGICAL EXAMS | 10.00 € | m190.22.3 | 1 | 1 | 1 | 1 | 0 | 0 | 0 | 0 | 0 | 0 | 1 | 1 | 1 | 1 | 1 | 1 | 0 |
| Iron | HAEMATOLOGICAL EXAMS | 2.00 € | m190.22.5 | 0 | 1 | 1 | 1 | 0 | 0 | 0 | 0 | 0 | 0 | 0 | 0 | 0 | 0 | 0 | 0 | 0 |
| Fibrinogen | HAEMATOLOGICAL EXAMS | 14.00 € | m190.64.5 | 1 | 0 | 1 | 1 | 0 | 0 | 0 | 0 | 0 | 0 | 1 | 1 | 1 | 1 | 1 | 1 | 0 |
| Leukocyte formula | HAEMATOLOGICAL EXAMS | 5.00 € | m190.70.4 | 1 | 1 | 1 | 1 | 0 | 0 | 0 | 0 | 0 | 0 | 1 | 1 | 1 | 1 | 1 | 1 | 0 |
| Alkaline phosphatase | HAEMATOLOGICAL EXAMS | 2.00 € | m190.23.5 | 1 | 1 | 1 | 1 | 0 | 0 | 0 | 0 | 0 | 0 | 1 | 1 | 1 | 1 | 1 | 1 | 0 |
| Inorganic phosphorus | HAEMATOLOGICAL EXAMS | 1.00 € | Rm190.24.5 | 1 | 1 | 1 | 1 | 0 | 0 | 0 | 0 | 0 | 0 | 0 | 0 | 0 | 0 | 0 | 1 | 0 |
| Gamma glutamyl transferase | HAEMATOLOGICAL EXAMS | 2.00 € | m190.25.5 | 1 | 0 | 1 | 1 | 0 | 0 | 0 | 0 | 0 | 0 | 1 | 1 | 1 | 1 | 1 | 1 | 0 |
| Glucose | HAEMATOLOGICAL EXAMS | 2.00 € | m190.27.1 | 1 | 1 | 1 | 1 | 0 | 0 | 0 | 0 | 0 | 0 | 1 | 1 | 1 | 1 | 1 | 1 | 0 |
| Blood group | HAEMATOLOGICAL EXAMS | 8.00 € | Hm190.65.3 | 1 | 0 | 1 | 1 | 0 | 0 | 0 | 0 | 0 | 0 | 0 | 0 | 0 | 0 | 0 | 0 | 0 |
| Bicarbonates (HCO3) | HAEMATOLOGICAL EXAMS | 1.00 € | m190.10.2 | 0 | 0 | 1 | 1 | 0 | 0 | 0 | 0 | 0 | 0 | 0 | 0 | 0 | 0 | 0 | 0 | 0 |
| Immunoglubulin A | HAEMATOLOGICAL EXAMS | 6.00 € | m190.69.4 | 1 | 0 | 1 | 1 | 0 | 0 | 0 | 0 | 0 | 0 | 1 | 1 | 1 | 1 | 1 | 1 | 0 |
| Immunoglubulin G | HAEMATOLOGICAL EXAMS | 6.00 € | m190.69.4 | 1 | 0 | 1 | 1 | 0 | 0 | 0 | 0 | 0 | 0 | 1 | 1 | 1 | 1 | 1 | 1 | 0 |
| Immunoglubulin M | HAEMATOLOGICAL EXAMS | 6.00 € | m190.69.4 | 1 | 0 | 1 | 1 | 0 | 0 | 0 | 0 | 0 | 0 | 1 | 1 | 1 | 1 | 1 | 1 | 0 |
| Interleukin 10, 12P70, 1BETA, 6, 8, TNFALFA | HAEMATOLOGICAL EXAMS | 21.60 € | Rm190.70.2 | 0 | 0 | 1 | 0 | 0 | 0 | 0 | 0 | 0 | 0 | 0 | 0 | 0 | 0 | 0 | 0 | 0 |
| Lactate dehydrogenase | HAEMATOLOGICAL EXAMS | 2.00 € | m190.29.2 | 1 | 1 | 1 | 1 | 0 | 0 | 0 | 0 | 0 | 0 | 1 | 1 | 1 | 1 | 1 | 1 | 0 |
| Lipase | HAEMATOLOGICAL EXAMS | 4.00 € | m190.30.2 | 1 | 1 | 1 | 1 | 0 | 0 | 0 | 0 | 0 | 0 | 0 | 0 | 0 | 0 | 0 | 1 | 0 |
| Magnesium | HAEMATOLOGICAL EXAMS | 2.00 € | m190.32.5 | 1 | 1 | 1 | 1 | 0 | 0 | 0 | 0 | 0 | 0 | 0 | 1 | 0 | 0 | 0 | 1 | 0 |
| C reactive protein | HAEMATOLOGICAL EXAMS | 5.00 € | m190.72.3 | 1 | 0 | 1 | 1 | 0 | 0 | 0 | 0 | 0 | 0 | 1 | 1 | 1 | 1 | 1 | 1 | 0 |
| Blood pH | HAEMATOLOGICAL EXAMS | 8.00 € | m190.36.2 | 0 | 0 | 1 | 1 | 0 | 0 | 0 | 0 | 0 | 0 | 0 | 0 | 0 | 0 | 0 | 0 | 0 |
| Potassium | HAEMATOLOGICAL EXAMS | 2.00 € | m190.37.4 | 1 | 1 | 1 | 1 | 0 | 0 | 0 | 0 | 0 | 0 | 1 | 1 | 1 | 1 | 1 | 1 | 0 |
| Total Proteins | HAEMATOLOGICAL EXAMS | 5.00 € | m190.39.1 | 1 | 1 | 1 | 1 | 0 | 0 | 0 | 0 | 0 | 0 | 1 | 1 | 1 | 1 | 1 | 1 | 0 |
| Oxygen saturation | HAEMATOLOGICAL EXAMS | 23.00 € | I89.7b.9 | 0 | 0 | 1 | 1 | 0 | 0 | 0 | 0 | 0 | 0 | 0 | 0 | 0 | 0 | 0 | 0 | 0 |
| Transferrin saturation | HAEMATOLOGICAL EXAMS | 6.00 € | m190.42.5 | 0 | 0 | 1 | 1 | 0 | 0 | 0 | 0 | 0 | 0 | 0 | 0 | 0 | 0 | 0 | 0 | 0 |
| Sodium | HAEMATOLOGICAL EXAMS | 2.00 € | m190.40.4 | 1 | 1 | 1 | 1 | 0 | 0 | 0 | 0 | 0 | 0 | 1 | 1 | 1 | 1 | 1 | 1 | 0 |
| Lymphocyte typing | HAEMATOLOGICAL EXAMS | 18.80 € | Rm190.81.5 | 1 | 0 | 1 | 1 | 0 | 0 | 0 | 0 | 0 | 0 | 1 | 1 | 1 | 1 | 1 | 1 | 0 |
| Transferrin | HAEMATOLOGICAL EXAMS | 4.00 € | Rm190.42.4 | 0 | 1 | 1 | 1 | 0 | 0 | 0 | 0 | 0 | 0 | 0 | 0 | 0 | 0 | 0 | 0 | 0 |
| Triglycerides | HAEMATOLOGICAL EXAMS | 2.00 € | m190.43.2 | 1 | 1 | 1 | 1 | 0 | 0 | 0 | 0 | 0 | 0 | 1 | 1 | 1 | 1 | 1 | 1 | 0 |
| Urea | HAEMATOLOGICAL EXAMS | 2.00 € | m190.44.1 | 1 | 1 | 1 | 1 | 0 | 0 | 0 | 0 | 0 | 0 | 1 | 1 | 1 | 1 | 1 | 1 | 0 |
| Erythrocyte sedimentation rate | HAEMATOLOGICAL EXAMS | 2.00 € | m190.82.5 | 1 | 0 | 1 | 1 | 0 | 0 | 0 | 0 | 0 | 0 | 1 | 1 | 1 | 1 | 1 | 1 | 0 |
| Venous blood sampling | HAEMATOLOGICAL EXAMS | 8.37 € |  | 1 | 1 | 1 | 1 | 0 | 0 | 0 | 0 | 0 | 0 | 1 | 1 | 1 | 1 | 1 | 1 | 0 |

^a^Only once.

CMV = Cytomegalovirus; CT = computed tomography; HCV = hepatitis C virus; HR = high resolution; ig = immunoglobulin; PET = positron emission tomography; 1 = Yes; 0 = No.

**Table S2. Link table between time point, phase of CAR T-cells pathway and stage.**

| **Time point** | **Phase of CAR T-cells pathway** | **Stage** |
| --- | --- | --- |
| 1 | Pre-leukapheresis | Stage 1 |
| 2 | Pre bridging therapy cycle | Stage 1 |
| 3 | Every day during hospitalization | Stage 1 or Stage 2 or Stage 3 |
| 4 | Pre-lymphodepletion | Stage 1 |
| 5 | Pre-hospitalization for infusion | Stage 1 |
| 6 | 1^st^ day after infusion | Stage 2 |
| 7 | 3^rd^ day after infusion | Stage 2 |
| 8 | 7^th^ day after infusion | Stage 2 |
| 9 | 10^th^ day after infusion | Stage 2 |
| 10 | 15^th^ day after infusion | Stage 2 |
| 11 | 21^st^ day after infusion (or at discharge) | Stage 2 |
| 12 | 1^st^ month after infusion | Stage 2 |
| 13 | 3^rd^ month after infusion | Stage 3 |
| 14 | 6^th^ month after infusion | Stage 3 |
| 15 | 12^th^ month after infusion | Stage 4 |
| 16 | Every day during CRS | Stage 2 |
| 17 | Every day during ICANS | Stage 2 |

CAR = chimeric antigen receptor; CRS = cytokine release syndrome; ICANS = immune effector cell-associated neurotoxicity syndrome.

**Table S3. Time-person spent per product, type of patient and stage.**

| Product | Procedures | Infused | Not infused | Failed leukapheresis | lymphocytes never sent | all 1 time | credit note sent | Stage | People | Time/Patients (hours) |
| --- | --- | --- | --- | --- | --- | --- | --- | --- | --- | --- |
| Tisa-Cel | Bag pickup at the blood bank | 1 | 1 | 1 | 1 | 0 | 0 | I | Health Care assistant | 0.5 |
| Tisa-Cel | Transfer bag transfer, sample collection for blood count and CD3 | 1 | 1 | 1 | 1 | 0 | 0 | I | Laboratory staff | 0.33 |
| Tisa-Cel | CD3 assessment | 1 | 1 | 0 | 1 | 0 | 0 | I | Laboratory staff | 1.5 |
| Tisa-Cel | Bag centrifugation and cryopreservation preparation | 1 | 1 | 0 | 1 | 0 | 0 | I | Laboratory staff | 0.75 |
| Tisa-Cel | Cryopreservation with planer | 1 | 1 | 0 | 1 | 0 | 0 | I | Laboratory staff | 1.5 |
| Tisa-Cel | Dry shipper setup for shipment to the pharmaceutical company | 1 | 1 | 0 | 0 | 0 | 0 | I | Laboratory staff | 0.75 |
| Tisa-Cel | Reception dry shipper with CAR T product | 1 | 1 | 0 | 0 | 0 | 0 | II | Laboratory staff | 0.75 |
| Tisa-Cel | Reception dry shipper with CAR T product | 1 | 1 | 0 | 0 | 0 | 0 | II | Pharmacist | 0.75 |
| Tisa-Cel | Thawing for reinfusion with 1 bag | 1 | 0 | 0 | 0 | 0 | 0 | II | Laboratory staff | 0.5 |
| Axi-Cel | Nanocool arrival and control | 1 | 1 | 0 | 0 | 0 | 0 | II | Laboratory staff | 0.5 |
| Axi-Cel | Delivery of documentation to the transfusion | 1 | 1 | 1 | 1 | 0 | 0 | I | Health Care assistant | 0.33 |
| Axi-Cel | Bag pickup at the blood bank | 1 | 1 | 1 | 1 | 0 | 0 | I | Health Care assistant | 0.5 |
| Axi-Cel | Mononuclear cells shipping and documentation management on management systems and portals | 1 | 1 | 0 | 0 | 0 | 0 | I | Laboratory staff | 1 |
| Axi-Cel | Reception dry shipper with CAR T product | 1 | 1 | 0 | 0 | 0 | 0 | II | Laboratory staff | 0.75 |
| Axi-Cel | reception dry shipper CAR T product | 1 | 1 | 0 | 0 | 0 | 0 | II | Pharmacist | 0.75 |
| Axi-Cel | Thawing for reinfusion with 1 bag | 1 | 0 | 0 | 0 | 0 | 0 | II | Laboratory staff | 0.5 |
| Both | Apheresis meeting | 0 | 0 | 0 | 0 | 0 | 0 |  | CAR T SPECIALIST | 1 |
| Both | Apheresis meeting | 0 | 0 | 0 | 0 | 0 | 0 |  | Physician | 1 |
| Both | Regional listing | 0 | 0 | 0 | 0 | 1 | 0 | I | Physician | 0.25 |
| Both | Knowledge interview and signing of informed consent | 0 | 0 | 0 | 0 | 1 | 0 | I | CAR T SPECIALIST | 1 |
| Both | Knowledge interview and signing of informed consent | 0 | 0 | 0 | 0 | 1 | 0 | I | Physician | 1 |
| Both | Pharmaceutical company portal entry | 1 | 1 | 0 | 0 | 0 | 0 | I | Physician | 0.25 |
| Both | AIFA entry and pharmacy file submission | 1 | 1 | 0 | 0 | 0 | 0 | I | Physician | 0.25 |
| Both | Lab-apheresis-department-pharmacy coordination | 0 | 0 | 0 | 0 | 1 | 0 | I | CAR T SPECIALIST | 1 |
| Both | Lab-apheresis-department-pharmacy coordination | 0 | 0 | 0 | 0 | 1 | 0 | I | Physician | 1 |
| Both | Lab-apheresis-department-pharmacy coordination | 0 | 0 | 0 | 0 | 1 | 0 | I | Pharmacist | 1 |
| Both | Lab-apheresis-department-pharmacy coordination | 0 | 0 | 0 | 0 | 1 | 0 | I | Laboratory staff | 1 |
| Both | Application for apheresis export authorization | 1 | 1 | 0 | 0 | 0 | 0 | I | Physician | 0.33 |
| Both | Aphaeretic visit | 0 | 0 | 0 | 0 | 0 | 0 | I | Physician | Code regional schedule m89.7^a^ |
| Both | Update AIFA sheets | 1 | 0 | 0 | 0 | 0 | 0 | II/III/IV | Physician | 0.67 |
| Both | Sending infusion attestation to company | 1 | 0 | 0 | 0 | 0 | 0 | II | CAR T SPECIALIST | 0.25 |
| Both | Credit note with disposal note for laboratory | 0 | 0 | 0 | 0 | 0 | 1 | II | CAR T SPECIALIST | 0.5 |
| Both | Credit note with disposal note for laboratory | 0 | 0 | 0 | 0 | 0 | 1 | II | Physician | 0.5 |
| Both | Credit note with disposal note for laboratory | 0 | 0 | 0 | 0 | 0 | 1 | II | Pharmacist | 0.5 |

^a^For transfusion service the price of aphaeretic visit instead of cost-per-person was used.

AIFA = Italian Medicines Agency; Axi-cel = axicabtagene ciloleucel; Both= axi-cel and tisa-cel; Tisa-cel = tisagenlecleucel.

**Table S4. Job (tasks) and hospitalization costs (euros).**

| *JOB* |  |
| --- | --- |
| LAB STAFF | 52.90 €/h |
| HemaTologist | 68.03 €/h |
| Pharmacist | 51.21 €/h |
| Nurse | 31.59 €/h |
| CAR T specialist | 23.16 €/h |
| healthcare worker | 24.02 €/h |
|  |  |
| *department* |  |
| transplantation unit | 999.22 €/day |
| ICU | 1.854.09 € /day |
| Hematology WARD | 727.58 €/day |
| OUTPATIENT WARD |  |
| PRODUCTIVE FACTOR OUTPATIENT WARD |  |
| C1 - (1) drugs and other treatments | 209.53 €/day |
| C2 - (2) OTHER HEALTH GOODS | 56.44 €/day |
| C3 - (3) NON-HEALTH GOODS | 6.27 €/day |
| C7 - (7) OTHER HEALTH SERVICES | 37.55 €/day |
| C8 - (8) CONSULTING, TEMPORARY EMPLOYMENT, AND HEALTH CARE COLLABORATIONS | 5.77 €/day |
| C10 - (10) MAINTENANCE | 0.05 €/day |
| C11 - (11) TECHNICAL SERVICES (INCLUDING UTILITIES) | 46.74 €/day |
| C14 - (14) OTHER NON-HEALTH SERVICES/ADMINISTRATIVE COSTS | 0.07 €/day |
| C23 - (23) EQUIPMENT DEPRECIATION | 14.90 €/day |
| C26 - (26) OTHER COSTS. | 0.02 €/day |
| Total | 377.34 €/day |

ICU = Intensive care unit.

**Table S5. Cost (Euros) per year of leukapheresis.**

|  | 2019 (n = 2) | 2020 (n = 23) | 2021 (n = 37) | 2022 (n = 18) | P value^a^ |
| --- | --- | --- | --- | --- | --- |
| tranfusion bags | 0 | 54,820 | 38,128 | 4,660 | 0.27 |
| Day service | 0 | 15,754 | 19,868 | 14,041 | 0.21 |
| Transplantation ward | 56,955 | 534,582 | 671,475 | 218,829 | 0.20 |
| intensive care unit | 0 | 129,786 | 72,309 | 16,686 | 0.33 |
| hematology ward | 0 | 65,482 | 74,940 | 23,282 | 0.62 |
| Bridging therapy | 0 | 42,766 | 73,125 | 91,713 | 0.34 |
| profilaxys | 1,839 | 44,193 | 55,873 | 66,265 | 0.48 |
| steroids | 0 | 18,737 | 13,770 | 22,106 | 0.44 |
| Extra infective Aes within 30 days from infusion | 0 | 5,326 | 149,072 | 24,644 | 0.94 |
| extra infective Aes after 30 days from infusion | 56 | 0 | 583 | 0 | 0.37 |
| infective Aes within 30 days from infusion | 3,362 | 108,443 | 28,715 | 8,188 | 0.39 |
| infective Aes after 30 days from infusion | 0 | 8,317 | 7,022 | 55,082 | 0.38 |
| Total | 62,213 | 1,028,211 | 1,204,886 | 545,500 | 0.4 |

^a^the test is weighted for number of patients and events (Kruskal-Wallis’ test).
